# Nephrokeli, a Chinese Herbal Formula, May Improve IgA Nephropathy through Regulation of the Sphingosine-1-Phosphate Pathway

**DOI:** 10.1371/journal.pone.0116873

**Published:** 2015-01-29

**Authors:** Yifei Zhong, Ke Wang, Xianwen Zhang, Xiaofan Cai, Yiping Chen, Yueyi Deng

**Affiliations:** 1 Department of Nephrology, Longhua Hospital, Shanghai University of Traditional Chinese Medicine, Shanghai, China; 2 Surgical Research Institute of Traditional Chinese Medicine Combined with Western Medicine, Shuguang Hospital, Shanghai University of Traditional Chinese Medicine, Shanghai, China; University of Kentucky, UNITED STATES

## Abstract

Nephrokeli (NPKL) is a Chinese herbal formula that has been used to treat patients with IgA nephropathy (IgAN) for improvement of proteinuria and kidney injury. However, the mechanism remains unclear. Sphingosine-1-phosphate (S1P) and its receptors S1PR2 and S1PR3 are known to play an important role in kidney disease. Here, we tested whether NPKL is able to regulate the S1P pathway in the kidney of IgAN rats. Four groups of rats were included in the study: Control, IgAN, IgAN treated with losartan, and IgAN treated with NPKL. The IgAN model was generated by injection of bovine serum albumin and staphylococcus enterotoxin B. We found that IgAN rats had increased staining for proliferating cell nuclear antigen (PCNA) in the mesangial area and increased mRNA and protein levels of S1PR2 and S1PR3 in the kidney compared to control rats. Connective tissue growth factor (CTGF), a downstream growth factor in the S1P pathway, was also elevated in the kidney of IgAN rats. Treatment with either NPKL or losartan was able to reduce PCNA staining and the expression of both S1PR2 and S1PR3 in the kidney of IgAN rats. However, NPKL (but not losartan treatment) reduced the expression of CTGF in the kidney of IgAN rats. In addition, we treated rat mesangial cells with sera collected from either NPKL-treated rats or control rats and found that NPKL-serum was able to reduce S1P-induced mesangial cell proliferation and the expression of S1PR2/S1PR3 and CTGF. NPKL also attenuates expression of fibrosis, inflammation, and oxidative stress markers in the kidney of IgAN rats. Our studies provide the mechanism by which NPKL attenuates kidney injury in IgAN rats.

## Background

Immunoglobulin A nephropathy (IgAN) is one of the most common primary glomerular diseases in the world and has its highest prevalence in Asian and Pacific countries. Fully 15–20% of patients with biopsy-proved IgAN develop end-stage renal disease in 10 years and 20–30% do so in 20 years [1]. Because of the relatively young onset age of IgAN (in the second and third decade of life) patients with IgAN cause a huge health and financial burden to society [2, 3]. Histologically, IgAN displays characteristics of mesangial cell proliferation and extracellular matrix expansion [4]. Clinically, patients present a variety of manifestations from gross hematuria to proteinuria [5]. Although the mechanism of IgAN is complicated, the deposition of IgA1 on mesangial cells indicates a critical role of mesangial cell injury in the pathogenesis of IgAN [6].

Sphingolipid metabolites such as ceramide, sphingosine and sphingosine-1-phosphate (S1P) play important roles in disease due to their critical roles in regulating cell proliferation, differentiation, apoptosis, and migration [7]. S1P is synthesized by sphingosine kinase (SPK)-mediated phosphorylation of sphingosine and is degraded to phospholipid and phosphocholine by S1P lyase1 [3, 8]. S1P exerts its functions by binding to its cell surface receptors [9]. At present, five S1P receptors (S1PR1, S1PR2, S1PR3, S1PR4 and S1PR5) have been identified, and they were previously referred to as endothelial differentiation gene receptors EDG1, EDG5, EDG3, EDG6 and EDG8, respectively. They are G-protein coupled receptors and have cell-specific distribution, indicating that S1P may have distinct effects in different types of cells via specific receptors [10]. A variety of growth factors (platelet-derived growth factor (PDGF) and epidermal growth factor (EGF)), a cytokine (TNF-α), and a G-protein coupled receptor agonist (N-formyl-methionine-leucine-phenylalanine—fMLP) are known to promote S1P synthesis [11, 12, 13, 14]. We have previously reported that a Chinese herbal formula developed by Dr. Yiping Chen in our group referred to as Nephrokeli (NPKL) is an effective therapy in patients with IgAN. In the present study, we wished to investigate whether NPKL improves IgAN through regulation of the S1P pathway in a rat model of IgAN.

## Methods

### Materials

Bovine serum albumin was purchased from AMRESCO (Cat. NO. 0409A09). Staphylococcal enterotoxin B was purchased from the Institute of Microbiology and Epidemiology of Chinese Academy of Military Medical Sciences (Cat. NO. 0419). All other chemicals were purchased from Sigma Co. (Shanghai, CN). The proliferative cell nuclear antigen (PCNA) staining kit was purchased from Santa Cruz Biotechnology (Santa Cruz, CA, USA). Antibodies for beta-actin, S1PR2, S1PR3, and connective tissue growth factor (CTGF) were purchased from Abcam. Losartan was purchased from Merck Pharmaceutical Co., Ltd (Hangzhou, China, H20000371).

### Preparation and analysis of Nephrokeli (NPKL)

NPKL consists of a mixture of 10 g nvzhenzi, 10 g guijia, 10 g shanyao, 10 g shengpuhuang, 20 g mohanlian, 10 g baisu, 10 g cangzhu, 30 g yiyiren, 10 shengdi, and 30 g sheshechao and was prepared by Tianjiang Pharmaceutical Company as a dry powder (Jiangyin, China). The active components in NPKL were further analyzed using the following methods: One gram powder of NPKL was refluxed 30 min in 10 ml methanol for an extraction. The extract was filtered through a 0.45 μm membrane and then 2.0 μl of the filtrate was analyzed by liquid chromatography-mass spectrometry (LC-MS). Chromatography was performed on an Agilent-1260 Series (Agilent, USA) LC system equipped with a binary pump, an online degasser, an auto plate-sampler, and a thermostatically controlled column compartment. The columns were maintained at 35°C. The separation was carried out on an Agilent parashell C18 column (100 mm ×2.1mm i.d., 2.7µm; Agilent). The binary gradient elution system consisted of 0.1% formic acid in water (solvent A) and 0.1% formic acid in acetonitrile (solvent B) and separation was achieved using the following gradient: 0–3 min 3% B; 3–15 min 3–50% B; 15–25 min 50–90% B; 25–30 min 90% B. The composition then returned to initial conditions and maintained for three column volumes for equilibration. The flow rate was 0.35 mL·min^−1^ and the injection volume was 2 µL. Mass spectrometry was performed using an Agilent 6530 Quadrupole-time-of-flight (QTOF) mass spectrometer (Agilent Corp, USA) equipped with an electrospray ionization (ESI) interface, and was operated in positive ion mode with parameters set as follows: capillary voltage, 4.0 kV; skimmer, 65 V; OCT 1 RF Vpp, 750 V; pressure of nebulizer, 35 psig; drying gas temperature, 320°C; sheath gas temperature, 350°C. Nitrogen was used as sheath and drying gas at a flow rate of 11.0 L·min^−1^ and 5.0 L·min^−1^, respectively. MS spectra were collected at 2.0 spectra·s^−1^, and MS/MS spectra were collected at 1.0 spectra·s^−1^, with a medium isolation window (~4 m/z units) and the collision energy was set between 15 and 30 V according to the situation. The accurate-mass capability of the TOF analyzer allowed reliable confirmation of the identity of the detected constituents, normally with mass errors below 5 ppm in routine analysis. An external calibration solution (Agilent calibration solution A) was continuously sprayed in the ESI source of the QTOF system, employing the ions with *m/z* 121.0508 (purine) and 922.0098 (hexakisphosphazine) to recalibrate the mass axis ensuring mass accuracy and reproducibility throughout the chromatographic run. Data were collected in centroid mode and the mass range was set at *m/z* 100–1200 using the extended dynamic range.

### Animal studies using the Rat IgAN model

A total of 44 female SD rats weighing 180±10 g were purchased from Slack Animal Company (Shanghai, China) and housed in the animal facility of Shanghai National Medical University with a room temperature of 21–25^0^C and humidity of 40%–50%. The rats had free access to food and water. This study was approved by the Ethics Committee of Longhua Hospital for animal studies and was performed in accordance with Guiding Principles for the Care and Use of Laboratory Animals. A total of 44 SD rats were randomly divided into 4 groups with 11 rats in each group according to numbers picked up by the software SPSS 15.0. The four groups were: control, IgAN, IgAN treated with NPKL and IgAN treated with losartan (50 mg/d). Because there were two deaths in the rat IgAN model the number of rats in each group was 11 rats in control, 10 rats in IgAN, 11 rats in IgAN+NPKL and 10 rats in IgAN+losartan. The IgAN model was generated by intraperitoneal injection of bovine serum albumin and staphylococcal enterotoxin as described previously [15, 16]. Rats in the control and IgAN groups were given double-distilled water while rats in the IgAN+NPKL group were treated with NPKL at 10 g/kg/d. Rats in the IgAN+losartan group were treated with losartan at 80 mg/kg/d. All rats were treated daily for 28 days by oral gavage. At the end of the experiment, the rats were weighed and then anesthetized by ip injection of 45 mg/kg 1% barbital. Laparotomy was performed and blood was collected from IVC. Kidneys were perfused with saline through the left ventricle until the kidney turned pale. Left kidneys were saved at -80^0^C for real time PCR and western blotting. Right kidneys were used for histology and immunostaining. In order to collect sera from the rats for in vitro experiments, we fed 5 rats with vehicle and 5 rats with 100 g/kg/d NPKL for 3 days and the blood was collected from these rats via IVC as described above. The sera were obtained after centrifugation at 1500 rpm/min for 15 minutes at 4°C. Because the sera were diluted in medium at 1:10 for cell stimulation, we fed the rats NPKL at a concentration 10 times higher than was used to treat the IgAN rats. The concentration of NPKL used in vitro would therefore be similar to what was used to treat the IgAN rats.

### Immunohistochemical staining

Formalin-fixed kidneys were embedded in paraffin and 0.4 µm thick sections were used. The slides were stained with a PCNA staining kit according to the manufacturer’s protocol. The slides were also counterstained with Hematoxylin (Thermo Scientific). The number of PCNA-positive nuclear cells and the number of total nuclear cells per glomerulus in 10 observation fields were scored blindly by 2 investigators. The ratio of PCNA-positive cells versus total nuclear cells per glomerulus was calculated and expressed. Immunohistochemical staining for Type III collagen, macrophage marker (F3/80), MCP-1, and NOX4 were also performed as described [17]. The following antibodies were used including antibodies for type III collagen (Abcam), F4/80 (Ebiosicence), Monocyte chemoattractant protein-1 (MCP-1) (Abcam), and NADPH oxidase 4 (NOX4) (Abcam).

### Western blot analysis of S1PR2, S1PR3, and CTGF

Total protein was extracted from kidneys by homogenizing the kidney cortex in protein lysis buffer (10 mmol/L phosphate buffer, 250 mmol/L sucrose, 1 mmol/L EDTA, 0.1 mmol/L PMSF, and 0.1% tergitol, pH 7.5). After homogenization, the solution was centrifuged at 100,000 g at 4^0^C and the supernatant was used for western blot analysis. The protein concentrations were determined by the BCA protein assay (Pierce). Protein from each sample (30 µg) was loaded onto a 12% SDS-acrylamide gel. After electrophoresis, the proteins were transferred onto nitrocellulose membranes. The membranes were incubated in a PBS buffer with 1% BSA and different antibodies including beta-actin, S1PR2, S1PR3, and CTGF antibodies and then with a secondary antibody conjugated with horseradish peroxidase (HRP). Western blots were visualized by using an enhanced chemiluminescence kit according to the manufacturer’s instructions and exposed to X-ray film. Densitometry analysis for quantification was performed as described previously [18]. The ratio of the interested protein to β-actin was shown.

### Real time reverse transcriptase polymerase chain reaction (RT-PCR)

Total RNA was extracted from the kidney cortex by using Trizol reagent and RNA concentrations were determined by UV spectrometry. cDNAs were synthesized from 2 µg of total RNA in accordance with the manufacturer’s protocol (Invitrogen, USA). The primers and PCR conditions are listed in Table 1. The primers were designed and synthesized by ANDA Company (Guangzhou, China). Real-time RT-PCR was performed using a DNA thermal cycler (ABI Prism 7300) according to the manufacturer’s instruction.

**Table 1:** Sequences of primers and real-time PCR conditions.

**Gene**		**Primers**	**Tm (^0^C)**	**Product (bp)**
S1PR2	Forward	5'- ACTCAGCCATGTACCTGTTC-3'	51.4	120
	Reverse	5'- ACTGCAAGGGAGTTAAGGAC-3'		
S1PR3	Forward	5'- TGTCTCCAACAGTGTGGTTC-3'	51.8	169
	Reverse	5'- CAGCACATCCCAATCAGAAG-3'		
SPHK1	Forward	5'- TTCTGGAGGAGGCTGAGGTA-3'	53.3	155
	Reverse	5'- CATTAGCCCATTCACCACCT -3'		
SGPP2	Forward	5'- GGTGCAAAACCTCTCTCTGC-3'	53.4	174
	Reverse	5'- AGCAATCCCAAAAACCTGTG -3'		
CTGF	Forward	5'- AAGACCTGTGGGATGGGC-3'	56.8	193
	Reverse	5'- TGGTGCAGCCAGAAAGCTC-3'		
GAPDH	Reverse	5'- GGCATTGCTCTCAATGACAA-3'	50.9	223
	Reverse	5'- TGTGAGGGAGATGCTCAGTG-3'		

### Rat mesangial cell culture

Rat mesangial cells were obtained from Sciencell Research Laboratories and cultured in DMEM with 10% FBS according to the manufacturer’s instructions.

### MTT cell proliferation assay

Cell proliferation was assessed by using an MTT cell proliferation assay kit according to the manufacturer’s instructions.

### Statistics

The statistical significance of differences was assessed by employing the ANOVA and Fisher’s PLSD tests with SPSS 15.0. All measurements are expressed as the mean ± SEM. Differences were considered to be significant when p<0.05.

## Results

First we performed an analysis of NPKL as described in the methods. We identified 9 major active components (Table 2). Based on the previous studies, several active components are known to have anti-oxidative stress, anti-inflammation, and anti-proliferation effects (Table 2).

**Table 2:** Active components of SPKL based on the Mass Spectrometry analysis.

**Active components**	**Potential effects**	**References**
luteolin-7-O-β-D-glucopyranoside	Anti-oxidative stress	[32]
Luteolin	Anti-inflammation	[33]
2-atractylenolide; asterolide	Anti-inflammation	[34]
hinesol/ Naphtho	Unknown	
pureonebio	Unknown	
ligustroflavon / Specnuezhenide	Anti-oxidative stress	[35] [36]
Catalpol	Anti-inflammation and anti-proliferation	[37] [38]
asperuloside	Anti-inflammation	[39]
apigenin /apigenin-7-O-β-D-glucuronide	Anti-proliferation, anti-oxidative stress, and anti-inflammation	[40] [41] [42]

**Figure 1:**
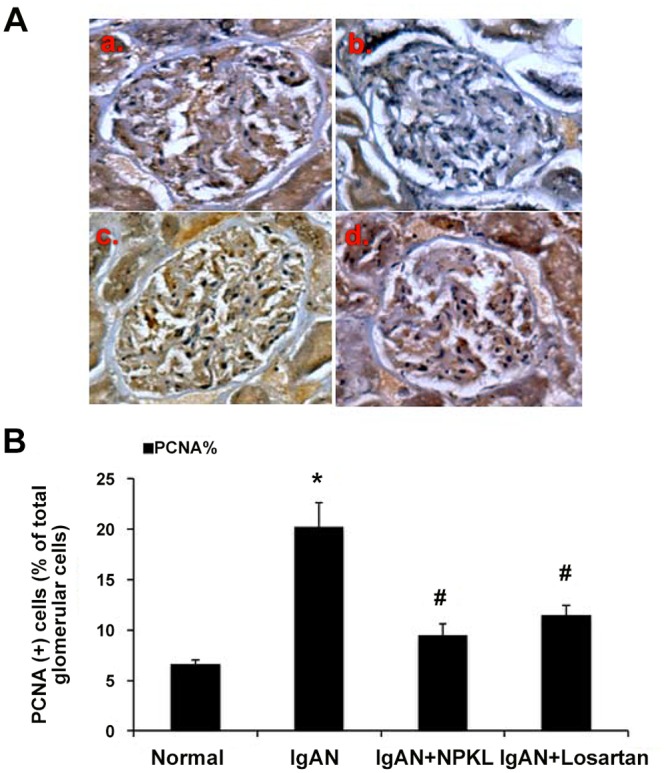
Immunohistochemical staining of PCNA in the glomerulus. Kidneys from the four different groups of rats were stained with antibody against PCNA and then counterstained with Hematoxylin to show nucleus. The top panel shows representative pictures of immunostaining of glomeruli in kidneys of normal rats (A), IgAN rats (B), IgAN+NPKL rats (C), and IgAN+losartan rats (D). The bottom panel shows quantitation of the PCNA staining, which is expressed as the mean±SD. *p<0.05 when compared with normal rats and ^#^p<0.05 when compared with IgAN rats, n = 10.

To test the effects of NPKL in IgAN, the rat IgAN model was successfully created in 31 of 33 rats that developed significant hematuria (93.94% successful rate). Two rats died during the creation of the IgAN model (6.06% failure rate). However, our rat IgAN model did not develop persistent hematuria or significant proteinuria. The evidence of IgAN in this model was confirmed by PCNA staining showing mesangial cell proliferation. As shown in Fig. 1, there was a significant increase in PCNA-positive nuclei in the glomerulus of kidneys with IgAN. However, treatment with NPKL or losartan reduced the number of PCNA-positive nuclei in the glomerulus, suggesting that both NPKL and losartan inhibit mesangial cell proliferation in these rats.

**Figure 2:**
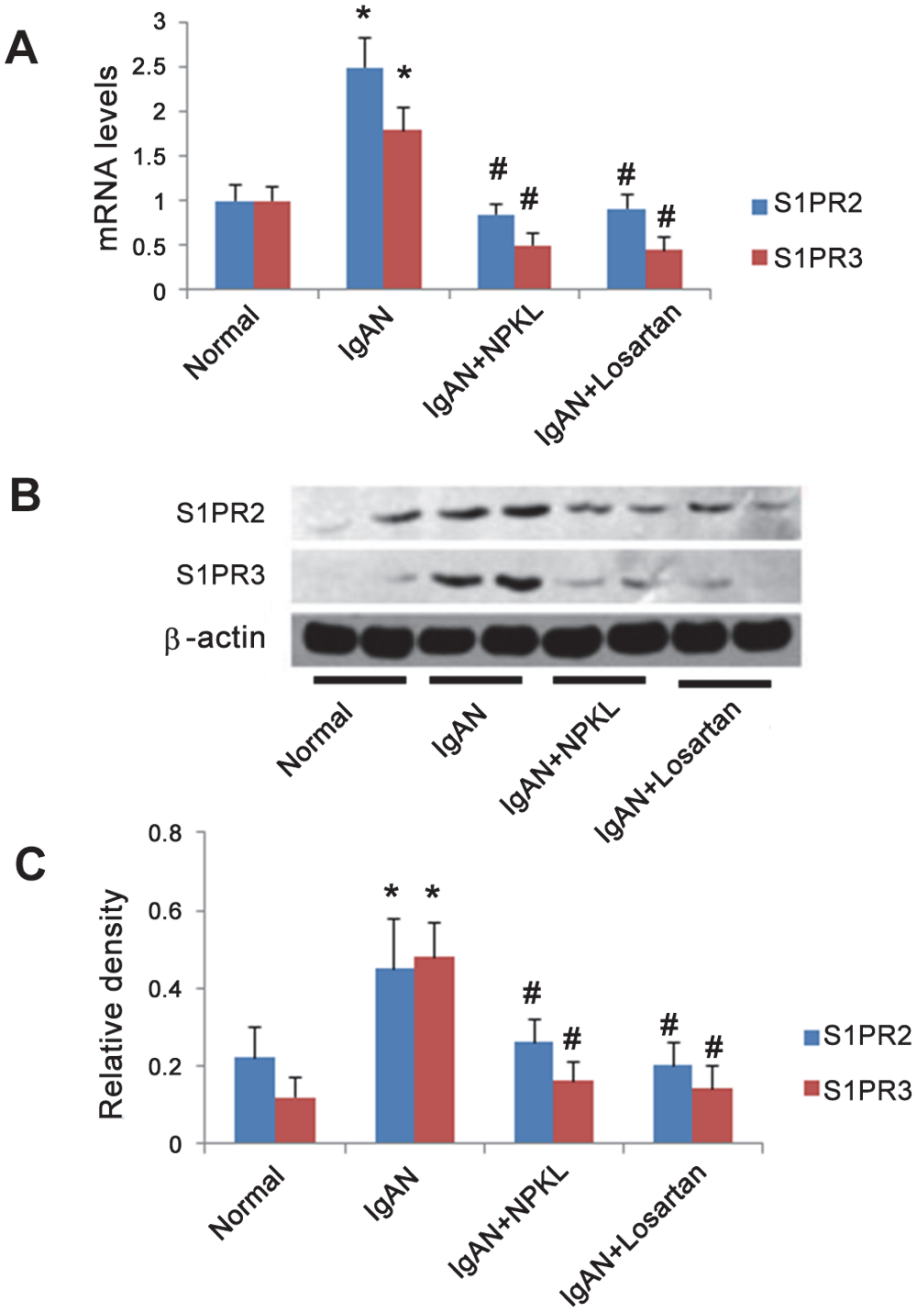
Regulation of S1PR2 and S1PR3 expression in the kidney of rats by NPKL. Total RNA and protein were extracted from the kidneys of rats in the different groups. Real time RT-PCR and western blotting were performed to examine S1PR2 and S1PR3 mRNA and protein levels. Panel A displays real time RT-PCR results. Panel B shows representative western blots of S1PR2 and S1PR3. Panel C shows the densitometric data of the western blots. The data are expressed as the mean±SD. *p<0.05 when compared with normal rats and ^#^p<0.05 when compared with IgAN rats, n = 10.

Because S1PR2 and S1PR3 are the key receptors mediating the effects of S1P in the kidney as previously reported [19], we asked whether NPKL and losartan affected their expression in the kidneys of these rats. As shown in Fig. 2A, we found that S1PR2 and S1PR3 mRNA levels were elevated in the kidney cortex of IgAN rats, whereas treatment with either NPKL or losartan caused a significant reduction of S1PR2 and S1PR3 mRNA levels in the kidneys of these IgAN rats. S1PR2 and S1PR3 protein levels showed a pattern of changes similar to those seen in Fig. 2B and 2C. These findings suggest that both losartan and NPKL inhibit the S1P pathway in the kidney by suppressing S1PR2 and S1PR3 expression.

**Figure 3:**
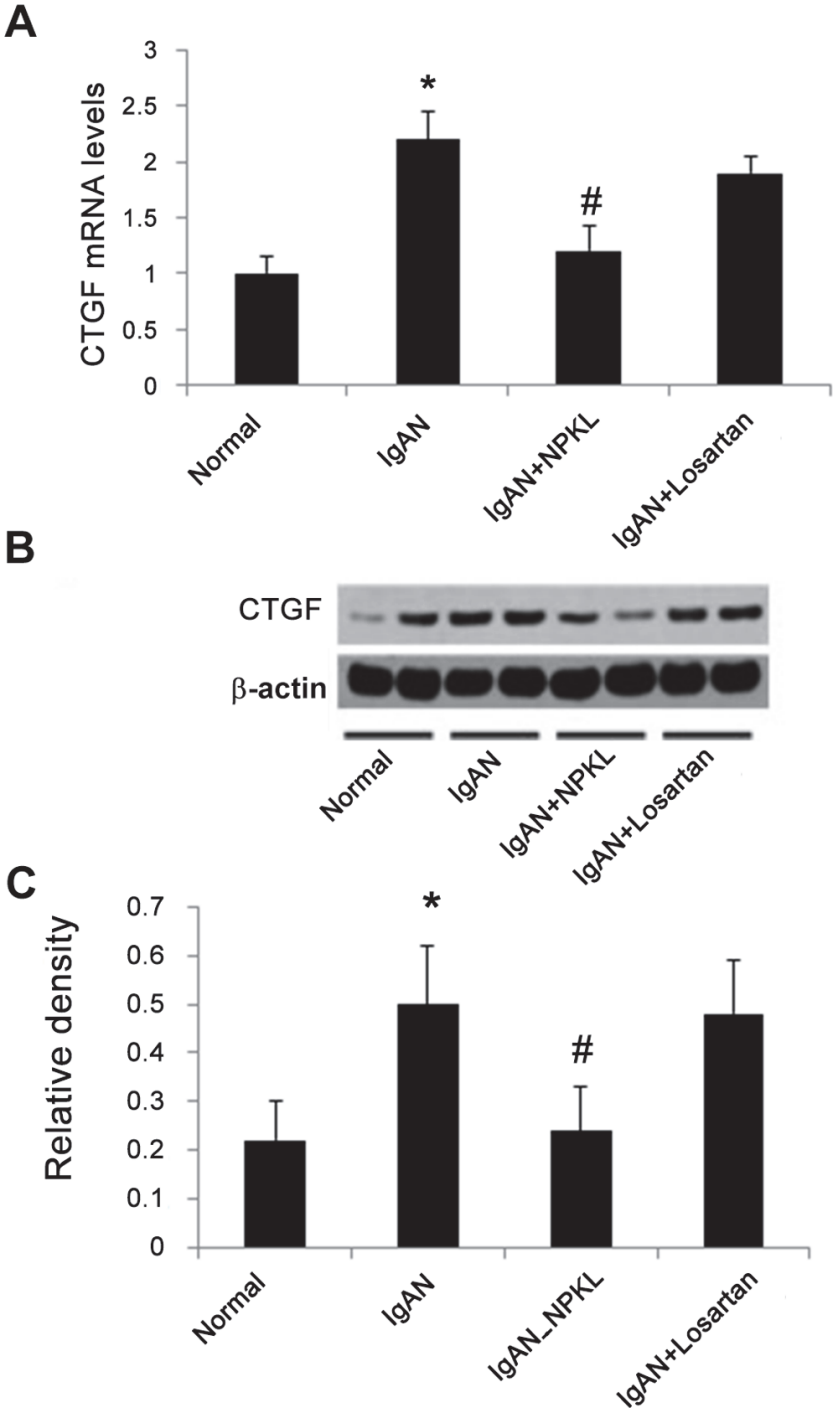
Expression of CTGF in the kidney. Real time RT-PCR and western blotting were performed to examine mRNA and protein levels of CTGF in the kidney of these rats. Panel A displays real-time RT-PCR results. Panel B shows representative western blots of CTGF. Panel C shows the densitometric data of the western blots. The data are expressed as the mean±SD. *p<0.05 when compared with normal rats and ^#^p<0.05 when compared with IgAN rats, n = 10.

Because CTGF has been shown to be up-regulated by S1P in mesangial cells [19], we further assessed the level of CTGF expression in the kidneys of these rats. As shown in Fig. 3, CTGF mRNA and protein levels were significantly elevated in the kidney cortex of IgAN rats. However, NPKL (but not losartan) treatment reduced the levels of CTGF mRNA and protein to normal.

**Figure 4:**
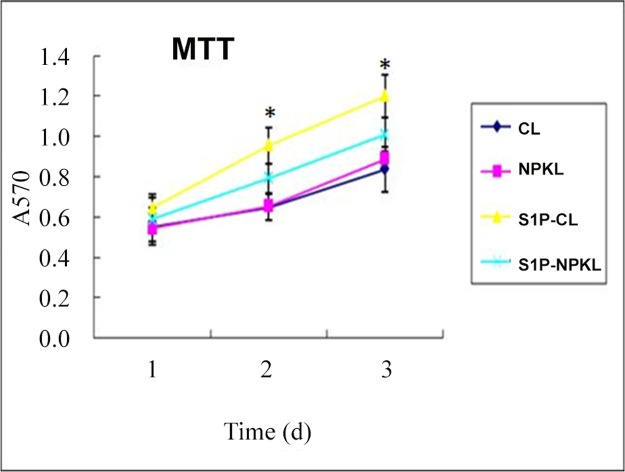
Effects of NPKL on mesangial cell proliferation. Mesangial cells were cultured and treated with vehicle or S1P together with sera from control rats (CL-Serum) or NPKL-treated rats (NPKL-Serum). Cell proliferation was assessed by MTT assay kit in these cells at days 1, 2 and 3. *p<0.01 when S1P-treated cells are compared with cells treated with both S1P and NPKL-Serum, n = 4.

**Figure 5:**
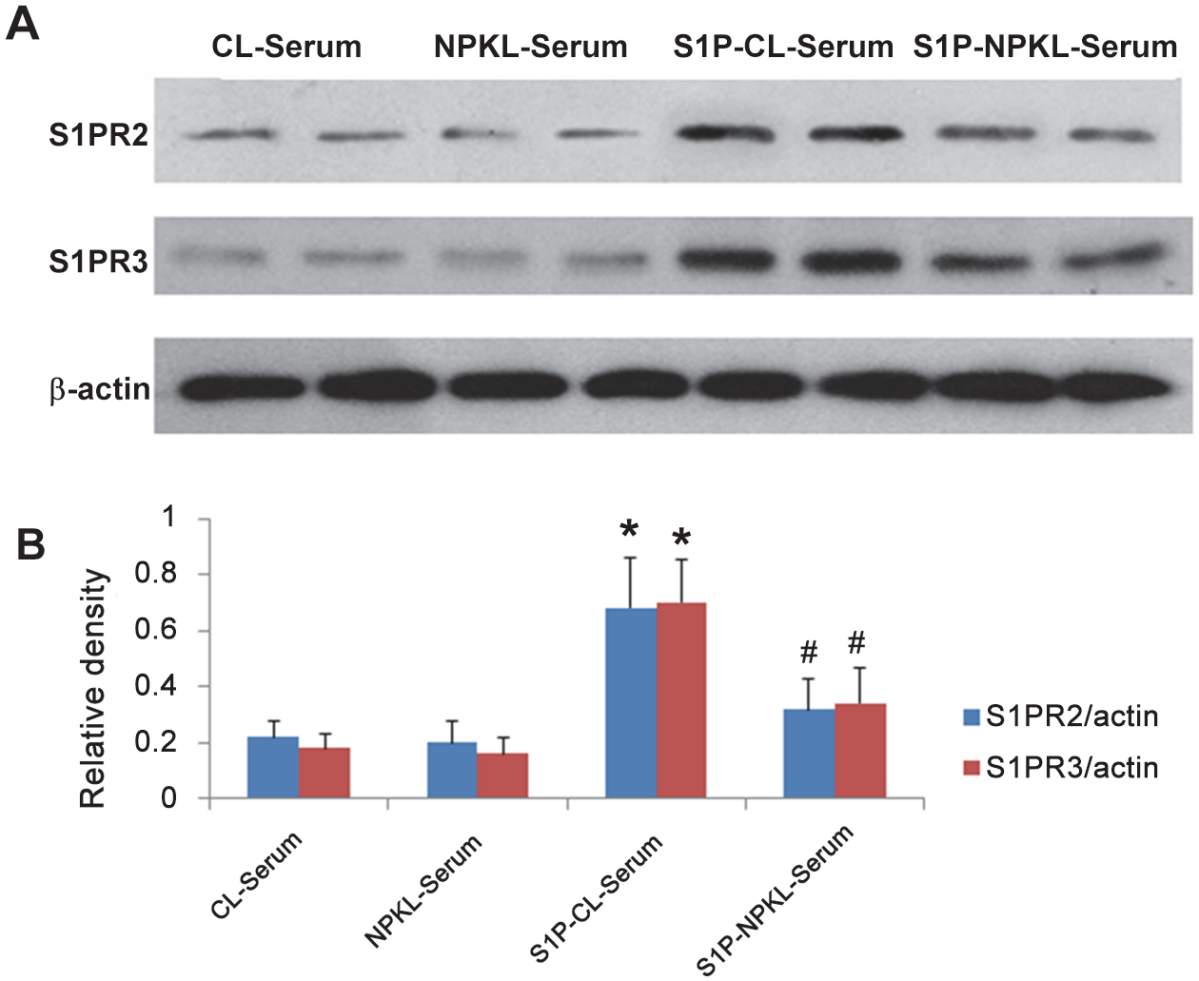
Effects of NPKL on the expression of S1PR2 and S1PR3 in mesangial cells. Mesangial cells were cultured and treated with vehicle or S1P together with sera from control rats (CL-Serum) or NPKL-treated rats (NPKL-Serum) for 24 hours and protein lysates were obtained from these cells for western blot analysis of S1PR2 and S1PR3. Representative western blots from three independent experiments are shown (A) and western blots were quantified by the densitometry (B). *p<0.05 when S1PR-CL-Serum are compared with CL-Serum and ^#^p<0.05 when S1PR-NPKL-Serum are compared with S1PR-CL-Serum.

**Figure 6:**
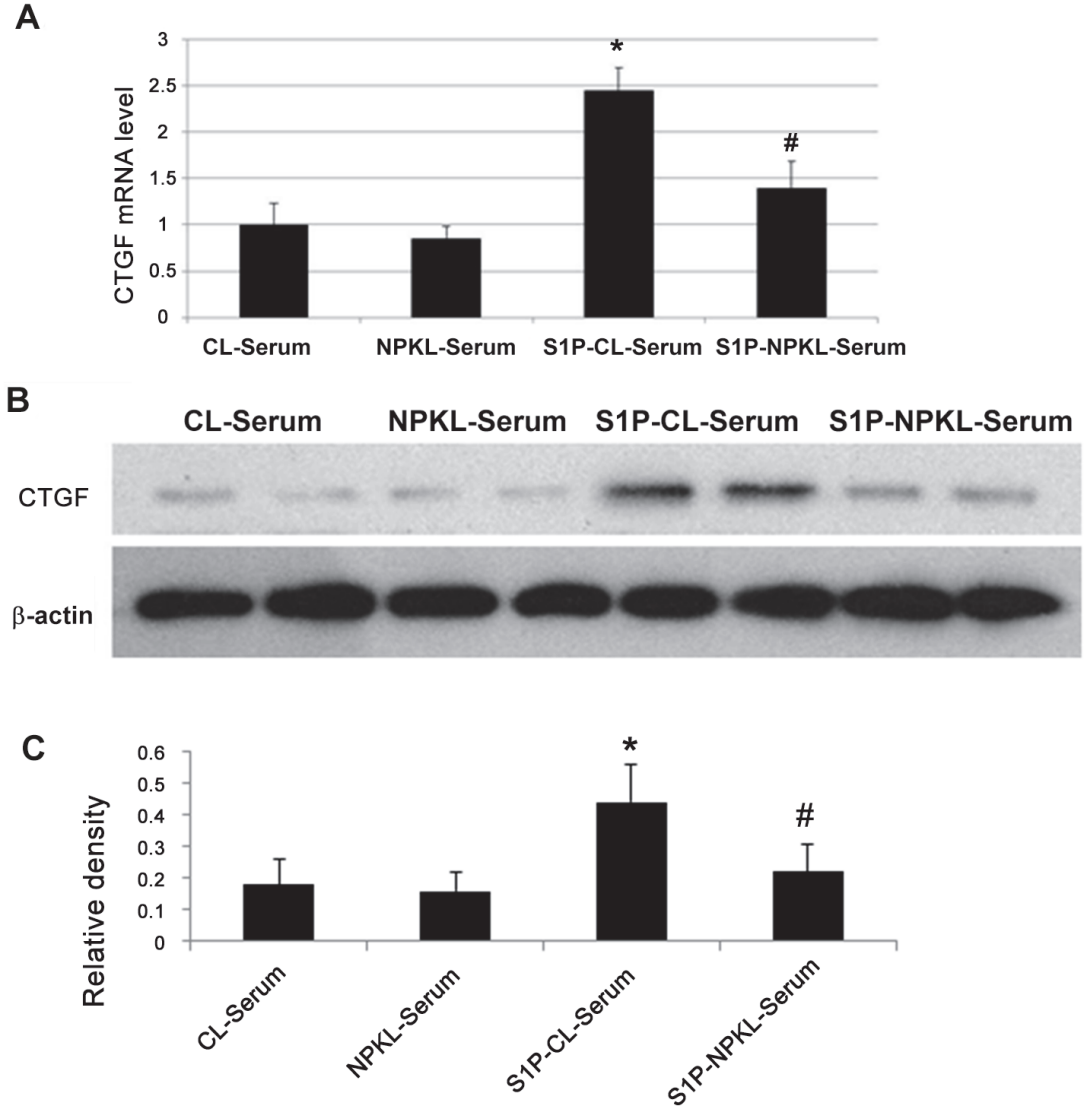
Effects of NPKL on the expression of CTGF in mesangial cells. Mesangial cells were cultured and treated with vehicle or S1P together with sera from control rats (CL-Serum) or NPKL-treated rats (NPKL-Serum) for 24 hours. Total RNA and protein lysates were obtained from these cells for real-time PCR analysis (A) and western blot analysis (B) of CTGF. Representative western blots from three independent experiments are shown. The western blots were analyzed by densitometry (C). *p<0.01 when compared with cells without S1P stimulation and ^#^p<0.05 when compared with cells stimulated with both S1P and NPKL-Serum, n = 3.

To determine the direct effects of NPKL in the regulation of the S1P pathway in mesangial cells, we used in vitro cultured rat mesangial cells. Chinese herbal medicines are usually taken after cooking in a large volume of water, and these herbal solutions are quite diluted. Therefore, it is difficult to add these Chinese herbal solutions directly to cultured cells. Therefore, sera collected from rats treated with either NPKL or vehicle were used to treat mesangial cells. This approach could help to assess whether the active components of NPKL in the sera of NPKL-treated rats affect mesangial cells. We found that the sera of NPKL-treated rats inhibited S1P-induced mesangial cell proliferation as assessed by MTT proliferation assay (Fig. 4) while no obvious cell toxicity was observed based on trypan blue staining (data not shown). We also found that the sera of NPKL-treated rats suppressed S1PR2/S1PR3 and CTGF expression in S1P-treated mesangial cells (Figs. 5–6).

**Figure 7:**
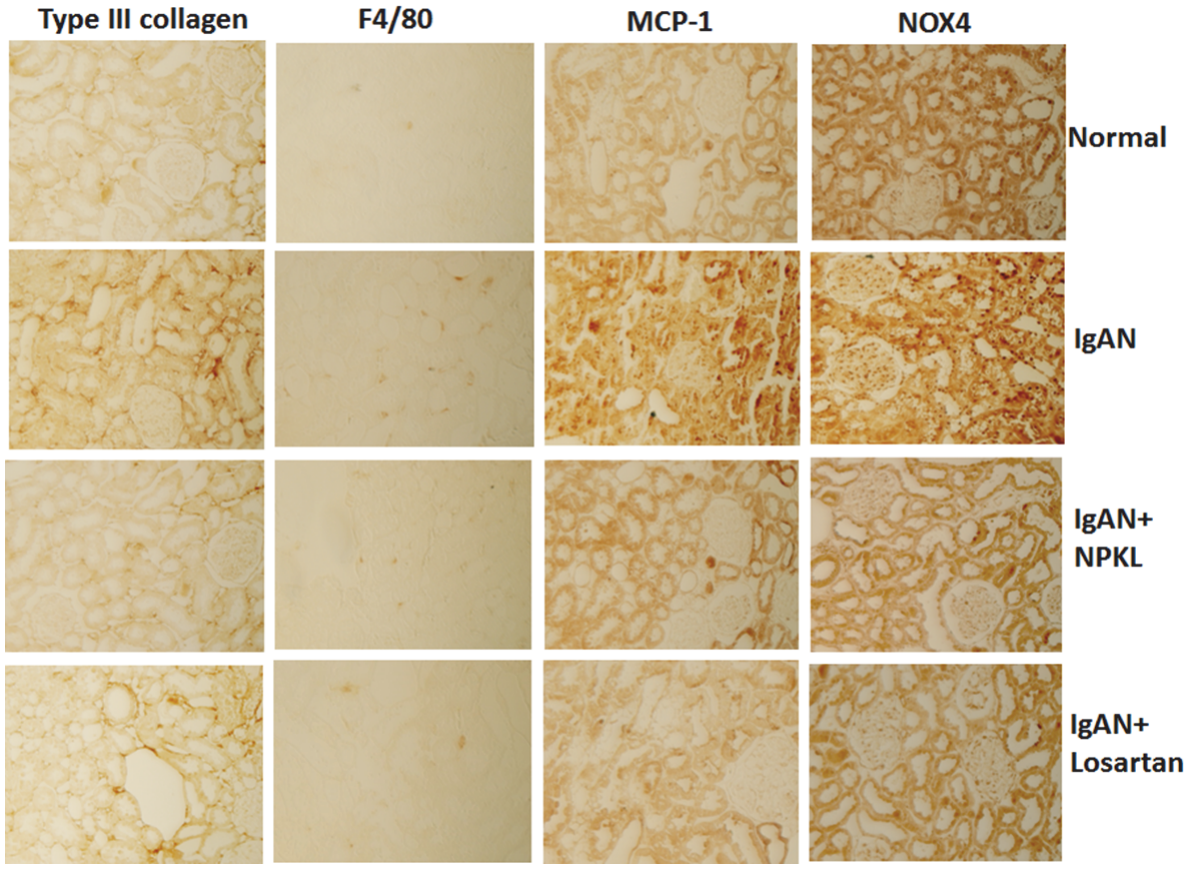
Effects of NPKL on the expression of fibrosis, inflammation, and oxidative stress markers in the kidneys of IgAN rats. Immunostaining was performed in the kidney sections of these rats using the specific antibodies as described in the method. The representative pictures of three different rats in each group are shown.

To determine whether NPKL also regulates other pathways to protect kidney injury in the IgAN rat, we examined expression of markers for fibrosis, inflammation, and oxidative stress. As shown in the Fig. 7, we found that the expression of Collagen III, F4/80 (macrophage marker), MCP-1, and NOX4 was increased in the IgAN kidney. However, the expression of these markers was markedly suppressed in the kidney of IgAN rats treated with either NPKL or losartan. These data suggest that, similar to losartan, NPKL could also attenuate fibrosis, inflammation, and oxidative stress in the kidney of IgAN rats.

## Discussion

Although the pathogenesis of IgAN is caused by multiple factors, including biochemical, immunological, and genetic factors the proliferation of mesangial cells in the glomerulus is a hallmark of IgAN. However, the exact mechanism of mesangial cell proliferation in IgAN remains unclear. Our study suggests that the S1P pathway may be involved in mesangial cell proliferation in IgAN.

Previous studies have suggested that S1P has significant effects in mesangial cells through binding to its receptors. It has been reported that low concentrations of advanced glycation endproducts promote S1P synthesis in the glomerulus, leading to glomerular mesangial cell hyperplasia in a diabetic kidney disease model [20]. S1P can bind to its receptors on mesangial cells to cause cellular hyperplasia and extracellular matrix deposition in the kidney [21, 22, 23]. A microarray study revealed a significant increase in S1PR2 and S1PR3, previously referred to as EDG3 and EDG5, in the kidney cortex of 6-week old HIGA mice [24]. That study also demonstrated that the specific receptors of S1P in glomerular mesangial cells are S1PR2 and S1PR3. S1P was found to be able to promote mesangial cell hyperplasia and proliferation by binding to S1PR2 and S1PR3. In addition, down-regulation of S1PR2 and S1PR3 by using antisense oligonucleotides reduces the anti-apoptotic effects of S1P in mesangial cells [25]. Moreover, a S1PR agonist (FTY720) stimulates S1PR3 activation in mesangial cells and results in activation of the transforming growth factor beta and Smad signaling pathways with up-regulation of CTGF and collagen [26]. Consistent with these findings, our study also suggests a key role of the S1P pathway in mesangial cell proliferation. In addition, we found that there was a significant increase in S1PR2 and S1PR3 expression in the kidneys of IgAN rats.

We report here that treatment with NPKL can inhibit the S1P pathway in IgAN rats and that this is a potential mechanism by which NPKL exerts its renal protective effects in patients with IgAN. NPKL is an empirical recipe developed by our group led by Dr. Yiping Chen in Longhua Hospital, and it has been used to treat patients with IgAN successfully over the last several years. NPKL consists of 9 Chinese herbs with kidney-nourishing properties based on traditional Chinese medicine theory. In a previous clinical study, we reported that NPKL is able to successfully reduce proteinuria in patients with IgAN [27]. Our current study suggests that NPKL may inhibit mesangial cell proliferation through regulation of S1PR2/S1PR3 expression in IgAN rats.

We also showed that both the mRNA and protein levels of CTGF were elevated in the kidneys of IgAN rats. CTGF is a peptide rich in cysteine that binds to extracellular matrix and heparin [28]. As a downstream effector of the transforming growth factor beta-1 (TGF-β1) pathway, CTGF induces expression of extracellular matrix proteins such as collagen, thereby contributing to kidney fibrosis [29, 30]. Because CTGF is up-regulated by activation of S1PR2 and S1PR3 [19], NPKL likely reduces CTGF expression through down-regulation of S1PR2 and S1PR3 expression in the kidneys of rats with IgAN. Interestingly, losartan did not affect CTGF expression in IgAN rats. The mechanism of this discrepancy between NPKL and losartan remains unclear. However, we speculate that NPKL may affect more pathways leading to CTGF expression than losartan does.

In addition, we confirmed that NPKL has direct effects in cultured mesangial cells: it suppresses S1P-induced mesangial cell proliferation, S1PR2/S1PR3 expression, and CTGF expression. We used sera from NPKL-treated rats to treat mesangial cells and observed the above effects. These findings provide evidence that the active compounds in the sera of NPKL-treated rats could be effective in mesangial cells.

Our data suggest that the renal protective effects of NPKL could be also through the regulation of other pathways including anti-inflammatory, anti-fibrosis, and anti-oxidative stress pathways. These effects are consistent with those reported previously with the active components that we identified from NPKL as shown in the table 2. It remains to be determined how S1P-induced signaling pathways crosstalk with the inflammatory and oxidative pathways. Additionally, NPKL may prevent kidney injury through other mechanisms that we have not explored yet.

We are aware of several limitations of the current study. First, NPKL is made of several herbal medicines. Through we have identified several active components of NPKL (Table 2) we don’t know which components are more effective than others. Interestingly, several active components that we identified are known to have anti-inflammation, anti-oxidation, and anti-proliferation effects and all these effects could mediate the renal protective effects of NPKL in the IgAN rat. In the future study, we will determine the effect of these individual components in the IgAN rat. However, it is also well-recognized that most Chinese herbal medications function as a formula containing several active compounds and a single active compound isolated from Chinese herbal formulae often did not work well [31]. Therefore, we could not predict whether individual active components isolated from NPKL could be as effective as NPKL. Another concern is that the rat model of IgAN does not completely mimic human IgAN. This rat model of IgAN does not develop persistent hematuria or significant proteinuria, and therefore we could not assess the effects of NPKL on these clinical phenotypes associated with IgAN even though NPKL attenuates proteinuria in patients with IgAN.

## Conclusion

Both in vitro and in vivo studies suggest an important role of the S1P pathway in mesangial cell injury. NPKL was able to attenuate mesangial cell proliferation in rats with IgAN, most likely through inhibition of S1PR2/S1PR3 expression. NPKL was able to reduce the expression of CTGF in cultured mesangial cells and the kidneys of IgAN rats. NPKL also attenuated expression of fibrosis, inflammation, and oxidative stress markers in the kidney of IgAN rats. Our study provides a potential mechanism by which NPKL prevents kidney injury in patients with IgAN.
